# Assessment of Prevalence and Diversity of Antimicrobial Resistant *Escherichia coli* from Retail Meats in Southern California

**DOI:** 10.3390/antibiotics12040782

**Published:** 2023-04-19

**Authors:** Katie Yen Lee, Kurtis Lavelle, Anny Huang, Edward Robert Atwill, Maurice Pitesky, Xunde Li

**Affiliations:** 1 Department of Population Health and Reproduction, School of Veterinary Medicine, University of California, Davis, Davis, CA 95616, USA; 2 Western Institute for Food Safety and Security, University of California, Davis, Davis, CA 95616, USA

**Keywords:** *Escherichia coli*, antimicrobial resistance (AMR), retail meat, phenotype, whole-genome sequencing (WGS), resistance genes, public health surveillance

## Abstract

Retail meat products may serve as reservoirs and conduits for antimicrobial resistance, which is frequently monitored using *Escherichia coli* as indicator bacteria. In this study, *E. coli* isolation was conducted on 221 retail meat samples (56 chicken, 54 ground turkey, 55 ground beef, and 56 pork chops) collected over a one-year period from grocery stores in southern California. The overall prevalence of *E. coli* in retail meat samples was 47.51% (105/221), with *E. coli* contamination found to be significantly associated with meat type and season of sampling. From antimicrobial susceptibility testing, 51 isolates (48.57%) were susceptible to all antimicrobials tested, 54 (51.34%) were resistant to at least 1 drug, 39 (37.14%) to 2 or more drugs, and 21 (20.00%) to 3 or more drugs. Resistance to ampicillin, gentamicin, streptomycin, and tetracycline were significantly associated with meat type, with poultry counterparts (chicken or ground turkey) exhibiting higher odds for resistance to these drugs compared to non-poultry meats (beef and pork). From the 52 *E. coli* isolates selected to undergo whole-genome sequencing (WGS), 27 antimicrobial resistance genes (ARGs) were identified and predicted phenotypic AMR profiles with an overall sensitivity and specificity of 93.33% and 99.84%, respectively. Clustering assessment and co-occurrence networks revealed that the genomic AMR determinants of *E. coli* from retail meat were highly heterogeneous, with a sparsity of shared gene networks.

## 1. Introduction

The emergence and dissemination of antimicrobial resistance (AMR) is of worldwide public health concern [1]. As a habitant of the endogenous microbiota of both humans and animals, *Escherichia coli* is both a commensal enteric bacterium and a pathogen responsible for various nosocomial, foodborne, and waterborne infections [2,3,4,5,6,7,8,9,10,11,12]. The increasing global incidence of multidrug resistant *E. coli*, particularly those resistant to therapeutically important drugs such as cephalosporins and fluoroquinolones, and last resort antibiotics such as carbapenem and colistin, prompts the need for integrated initiatives to monitor and reduce the spread of resistant organisms and their AMR genetic determinants [13,14,15,16]. Enhancing our understanding of AMR in *E. coli* is important as their ubiquity and genomic plasticity enables high frequency of AMR mobilization, which promotes the acquisition and transfer of resistance to other bacterial species [17,18]. Hence, commensal *E. coli* is also frequently utilized as indicator organisms of AMR for the broader microbial community [19,20,21].

Antimicrobial use in human and veterinary medicine are perceived as key drivers of AMR emergence [22,23], with selective pressures imposed amongst food animals comprising one avenue of public health concern [24,25]. Raw foods of animal origin such as meat products may serve as reservoirs and conduits for AMR [26,27], and have been included in integrated monitoring efforts due to their epidemiological linkage to foodborne outbreaks involving zoonotic pathogens [28,29] and the need to better understand the maintenance and dissemination of AMR along the food chain [30]. In the United States, the National Antimicrobial Resistance Monitoring System (NARMS) monitors AMR in various foodborne bacteria—including commensal *E. coli*—from humans, food-producing animals, and retail meat [31]. The distribution of AMR has been suggested to vary geographically [1,32,33], with human activity and movement of food and animals contributing to the evolution of bacterial populations [34,35,36,37].

In this study, we present the first available data on *E. coli* from retail meats in southern California as part of expanded surveillance coverage of NARMS retail meat sampling in 2018. The objective of this study was to determine the prevalence, the distribution of AMR and associated genetic determinants, and the potential drivers of AMR variability in *E. coli* from retail meat. This study aims to enhance our understanding of AMR in foodborne *E. coli*, and to provide insight on the clinical and epidemiologic risks associated with retail meat products in California. 

## 2. Results

### 2.1. Risk Factors Associated with the Presence of E. coli in Retail Meat Products

*Escherichia coli* isolates were recovered from 47.51% (105/221) of samples, with the highest frequency observed in ground turkey (70.37%, 38/54), followed by chicken (67.86%, 38/56), pork chop (32.14%, 18/56), and ground beef (20.00%, 11/55) (Table 1). 

From multivariable logistic regression, risk factors which were significantly associated with the presence of *E. coli* in retail meat products included meat type and season. The odds of *E. coli* isolation were 9.43 (95% CI 3.84–23.21) and 11.00 (95% CI 4.39–27.54) times higher in skin-on/bone-in chicken and ground turkey compared to ground beef products, respectively. Samples purchased in the spring, summer, and autumn had a 0.22 (95% CI 0.083–0.55), 0.36 (95% CI 0.14–0.91), and 0.36 (95% CI 0.14–0.91) times odds of *E. coli* isolation compared to those purchased in the winter. (Table 1). 

### 2.2. Phenotypic Antimicrobial Resistance of E. coli from Retail Meat Products

From antimicrobial susceptibility testing (AST) of 105 *E. coli* isolates, 51 (48.57%) were susceptible to all antimicrobials tested, 54 isolates (51.43%) were resistant to at least 1 drug, 39 (37.14%) to 2 or more, and 21 (20.00%) to 3 or more drugs. All isolates were susceptible to azithromycin and meropenem, and 15 (14.29%) were multidrug resistant (MDR). Of the MDR isolates, 10 (66.67%) were from ground turkey, 3 (20%) were from chicken, and 2 (13.33%) were from pork chops. 

*E. coli* isolates exhibited the highest overall frequency of resistance to tetracycline (43.81%), followed by streptomycin (30.48%), ampicillin (20.95%), and gentamicin (16.19%) (Table 2). From exact logistic regression, isolates from ground turkey had a significantly higher odds of resistance to ampicillin (OR 4.94, 95% CI 1.17–30.11), streptomycin (OR 3.81, 95% CI 1.10–15.56), and tetracycline (OR 5.24, 95% CI 1.65–18.55) compared to isolates from non-poultry meat types (beef and pork). Isolates from chicken and ground turkey products also exhibited higher odds of resistance to gentamicin compared to those from non-poultry origin; however, the association was only significant for isolates from chicken (OR 8.47, 95% 1.05–394.18) (Table 3). Collectively, *E. coli* isolates in this study had high diversity of phenotypic AMR patterns, with a total of 21 unique antibiogram patterns identified amongst the 54 non-susceptible isolates. Contributing to this diversity in distribution of antibiogram patterns were three isolates—two MDR isolates from pork chops and one non-MDR from ground turkey—with decreased susceptibility to ciprofloxacin (Table 4). 

### 2.3. Genetic Determinants of AMR in E. coli from Retail Meat Products

**Figure 1:**
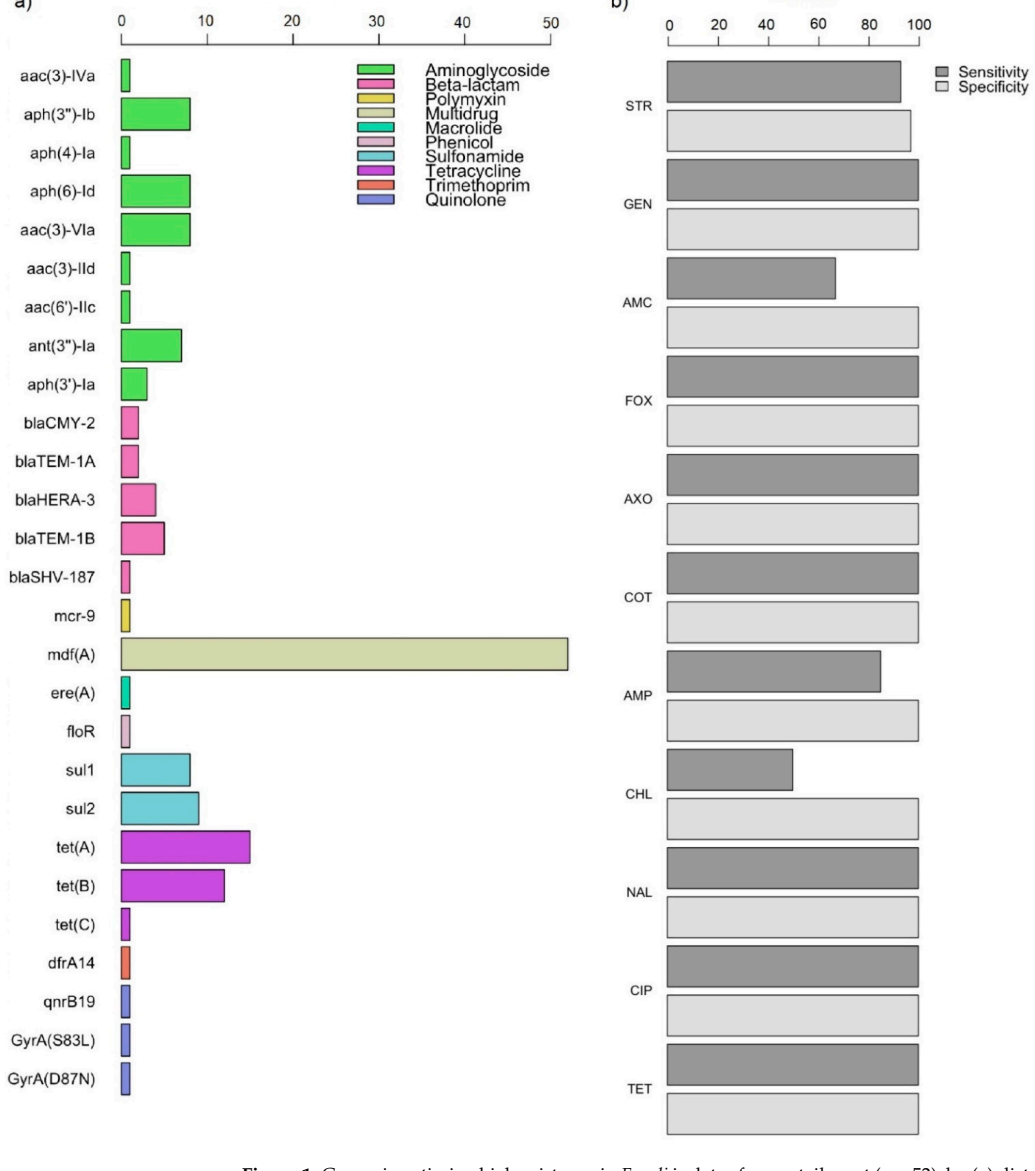
Genomic antimicrobial resistance in *E. coli* isolates from retail meat (n = 52), by (**a**) distribution of antimicrobial resistance genes and (**b**) concordance with phenotypic antimicrobial resistance. STR: streptomycin; GEN: gentamicin; AMC: amoxicillin/clavulanic acid; FOX: cefoxitin; AXO: ceftriaxone; COT: trimethroprim-sulfamethoxazole; AMP: ampicillin; CHL: chloramphenicol; NAL: nalidixic acid; CIP: ciprofloxacin; TET: tetracycline.

Whole-genome sequencing (WGS) was conducted on a random subset of *E. coli* isolates in this study (n = 52). The genomes of these 52 *E. coli* isolates were screened for AMR genetic determinants including point mutations conferring quinolone resistance. A total of 27 AMR determinants were identified, including *mdf(A)*, which was detected in all isolates, and other commonly occurring genes corresponding to tetracycline (*tet(A)*, *tet(B)*, and *tet(C)*) and sulfonamide (*sul1* and *sul2*) resistance. The largest diversity of genes was observed for aminoglycoside resistance (nine genes encoding for various acetyltransferases, nucleotidyltransferases, or phosphotransferases) followed by those for beta-lactam resistance (*bla*_CMY-2_, *bla*_TEM-1A_, *bla*_TEM-1B_, *bla*_HERA-3_, and *bla*_SHV-187_). Notably, a variant of a colistin resistance gene, *mcr-9*, was detected in one MDR *E. coli* isolate from a chicken sample. Quinolone resistance genetic determinants were detected in two isolates including (1) a plasmid-mediated quinolone resistance (PMQR) gene, *qnrB19*, from an MDR isolate from pork chop; and (2) two chromosomal mutations in *gyrA* encoding S83L and D87N amino acid substitutions from an MDR isolate from ground turkey. Other resistance genes detected included those conferring resistance to macrolide (*ere(A)*), phenicol (*floR*), and folate synthesis inhibitor (*dfrA14*) drug(s) (Figure 1a). 

### 2.4. Concordance of AMR Phenotype and Genotype

Based on the 52 *E. coli* isolates that underwent both WGS and AST, the drug-specific and overall sensitivity and specificity was determined to assess WGS predictions of resistant and susceptible AMR phenotypes, respectively. In this study, AMR genetic determinants identified through WGS predicted phenotypic AMR with an overall sensitivity of 93.33% and specificity of 99.84%. The greatest discordance for sensitivity was observed for chloramphenicol (50%), followed by amoxicillin/clavulanic acid (66.67%), ampicillin (84.62%), and streptomycin (93.33%). Discordance for specificity was observed only for streptomycin (97.30%) (Figure 1b, Table S3).

### 2.5. Clustering Analysis of E. coli Isolates

**Figure 2:**
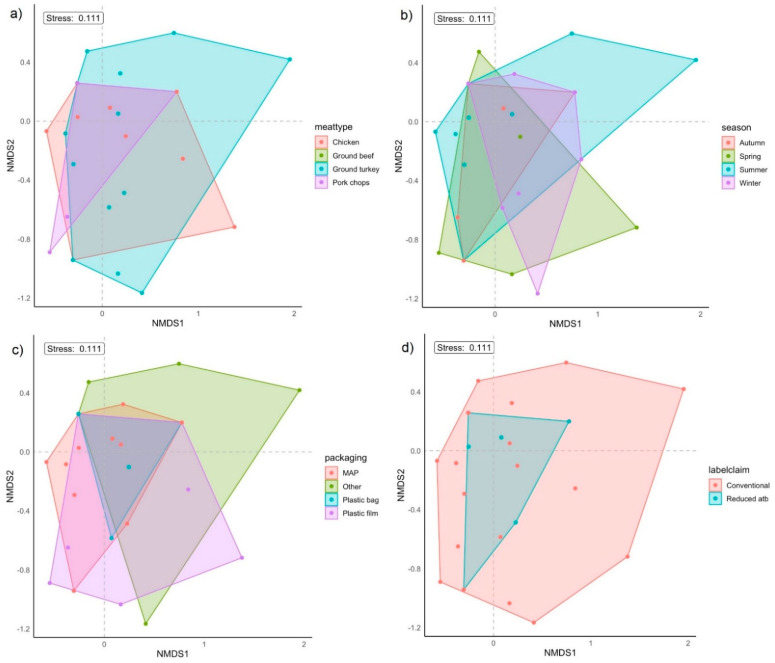
Non-metric multidimensional scaling (NMDS) performed using presence and absence of antimicrobial resistance genes from *E. coli* isolates from retail meat (n = 52). Individual points represent an isolate, with convex hulls displaying grouping factors of (**a**) meat type; (**b**) season; (**c**) packaging type; and (**d**) label claim.

From the presence and absence of AMR genetic determinants, *E. coli* isolates of retail meat origin in this study did not differ significantly by meat type, season, packaging type, and label claim (PERMANOVA and ANOSIM *p* > 0.05; Figure 2 and Table 5). Tests for differences in *E. coli* AMR genetic compositions indicated that dispersion differences were present among retail meat types (PERMDISP2 *p* < 0.05; Table 5). The results of PERMANOVA and/or ANOSIM by the grouping factor of meat type should thus be interpreted with care due to the assumption of equal variance among meat types not being met. By season, packaging type, and label claim, the compositional variance among the groups for each of these factors was not significantly different (PERMDISP2 *p* > 0.05; Table 5). Overall, the grouping factors assessed in this study each accounted for a relatively low proportion of the variance in *E. coli* AMR genetic composition (PERMANOVA R^2^ 0.0094–0.088), with indications of even distributions of ranks within and between groups for packaging types (ANOSIM R = 0.038) and greater dissimilarities in the average of ranks within-group than those of between-groups for meat type, season, and label claim (ANOSIM R < 0, Table 5) [38].

### 2.6. Co-Occurrence Networks of AMR Genetic Determinants in E. coli Isolates

**Figure 3:**
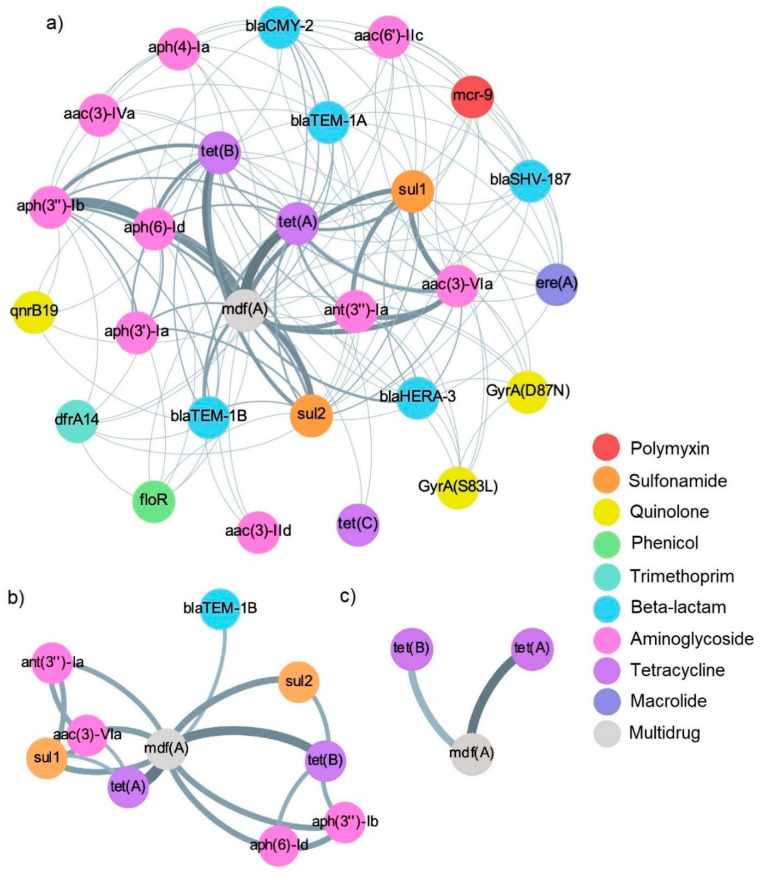
Co-occurrence networks of antimicrobial resistance genes in *E. coli* isolates (n = 52) by frequency thresholds of (**a**) 1 isolate; (**b**) 5 isolates; and (**c**) 10 isolates. Each node represents a gene and is color coded by antimicrobial class. Edges indicate frequency of co-occurrence, with a light to dark gradient representing low to high co-occurrence, respectively.

Despite the diversity in AMR genetic determinants present in *E. coli* isolates in this study (Figure 3a), the co-occurrence network of AMR genes was sparse, with the most commonly co-occurring genes being *mdf(A)* with *tet(A)* and *mdf(A)* with *tet(B)* at a co-occurrence frequency of 15 (28.85%) and 12 (23.08%) genomes, respectively (Figure 3c). At a lower co-occurrence threshold of ≥5 isolates, the networks of *mdf(A)* with *tet(A)* and/or *tet(B)* genes co-occurred with either a gene cluster comprised of *aph(3″)-Ib* and *aph(6)-Id* or another including *aac(3)-VIa*, *ant(3″)-Ia*, and *sul1* (Figure 3b). 

## 3. Discussion

The data presented in this study were collected from raw meat products from retail stores in southern California using the same sampling and processing protocols from FDA NARMS, thus enabling comparisons between our data and previous/concurrent NARMS data collected. No NARMS data have been available on *E. coli* from retail meats in California, as only certain pathogens of interest (*Salmonella* and *Campylobacter*) have been included in routine AMR assessment for California prior to this study.

Of the 221 retail meat products collected from southern California in 2018 and assessed in this study, *E. coli* was isolated from around half of the samples. Compared to the national average for NARMS retail testing for the same year, isolation of *E. coli* from samples in California was higher overall (47.51% vs. national average 28.75%) with higher recovery of *E. coli* in our study for chicken (67.86% vs. national average 19.22%), ground turkey (70.37% vs. national average 45.65%), and pork chop (32.14% vs. national average 25.82%), and lower recovery in ground beef (20.00% vs. national average 28.54%) [39].

Previous studies assessing the presence of enteric bacteria in meat products have found substantial variability in the prevalence of *E. coli* [26,40,41,42], which could be due to differences in sampling methodology, sample composition and origin, and processing protocols. Nevertheless, the significant association between *E. coli* contamination and meat type observed in our study is congruent with findings from studies conducted in multiple countries, where chicken and/or turkey products were found to have comparably higher prevalence of *E. coli* relative to non-poultry meat types such as beef and pork [26,41,42,43]. Additionally, season of retail meat purchase in our study was also significantly associated with the presence of *E. coli*, which could be explained by season serving as a surrogate for other unmeasured but correlated variables such as temperature, climate, and other temporal factors that more directly reflect the abiotic and biotic drivers of enteric bacteria persistence and proliferation in meat products [44]. This is substantiated by findings from a previous study which evaluated the relationship between weather variables and zoonotic foodborne pathogen contamination of meat products along the food chain in Canada. Although no definite seasonal trend was identified in their study for generic *E. coli*, Smith et al. found correlations between increased total precipitation and increased average/monthly temperature and *E. coli* in retail beef and pork, respectively [45].

The elevated frequencies of ampicillin, streptomycin, and tetracycline resistance in *E. coli* from ground turkey and gentamicin resistance in chicken compared to non-poultry meats (beef and pork) in our study are consistent with previous data collected in the United States [26,46,47]. While *E. coli* resistance to certain antimicrobials appears to be globally ubiquitous—for instance, high tetracycline resistance in *E. coli* from retail meats documented in Canada, India, Korea, and China—the overall heterogeneity across global frequencies of resistance is most likely attributed to differences in antimicrobial usage and the variability in selective pressures imposed across different countries and food animal production sectors [41,42,48,49]. For instance, a previous study reported 75.7% of *E. coli* from poultry meat in Korea being resistant to nalidixic acid, which, while consistent with national data of veterinary antibiotics sold for use in Korean poultry production [50], is in contrast to findings in our study of low nalidixic acid (1.90%) and ciprofloxacin (0.95%) resistance that are likely reflective of the ban of fluoroquinolone use in poultry production and restrictions in fluoroquinolone use for other food animal species in the US by the Food and Drug Administration [51,52].

The detection of *mcr-9*—a gene encoding a putative phosphoethanolamine transferase that reduces affinity for colistin—from an MDR *E. coli* isolate from retail chicken in this study is noteworthy as colistin is not used for treatment of food animals in the United States [53,54]. Colistin is a polymyxin antimicrobial and one of the few last-resort drugs available to treat life-threatening multidrug drug-resistant (MDR) and extensively drug-resistant (XDR) infections caused by Gram-negative bacteria such as carbapenem resistant *E. coli*, *P. aeruginosa*, and *K. pneumoniae* [55,56]. It was previously believed that colistin resistance was solely mediated by chromosomal genes (*phoPQ*, *pmrAB*, and *mgrB*) until the plasmid-mediated *mcr-1* gene from China was first reported in 2015 [57], and *mcr-1* variants (*mcr-2* through *10*) were subsequently identified in over two dozen bacterial species across six continents [58,59,60]. In the United States, *mcr-9* was first identified in a *Salmonella enterica* serotype Typhimurium clinical isolate in 2019 [61]. Since then, analysis of previously collected bacterial isolates from routine NARMS retail meat surveillance has traced *mcr-9* in isolates collected as far back as 2002, with findings of its occurrence in a pronounced proportion of *Salmonella* isolates in the US (28.6%, 2002–2019)—particularly *S.* Saintpaul from ground turkey—and also in a few *E. coli* isolates from samples collected in 2018 and 2019 [54]. Epidemiologically, *mcr-9* has not been linked to clinical resistance of colistin [54,61], which is consistent with findings here of *mcr-9* carriage in a colistin-susceptible *E. coli* isolate. It has, however, been shown that the presence of sub-inhibitory levels of colistin is sufficient to induce *mcr-9* expression through mediation by the two-component regulatory system *qseBC*, resulting in elevated MIC levels [53]. This highlights the importance of bacterial genomic surveillance efforts as disseminated *mcr* genes and other resistant determinants of high public health concern can remain undetected and/or unexpressed in non-resistant isolates until induced by antimicrobial exposure [53].

Consistent with the diversity of phenotypic AMR profiles of *E. coli* isolates, whole-genome sequencing identified several other AMR genetic determinants, including those mediating resistance to other drugs of high clinical importance such as fluoroquinolones and cephalosporins. Genetic characterization of fluoroquinolone resistance has been well documented to occur through combinations of chromosomal mutations within DNA gyrase (e.g., *gyrA*) and/or topoisomerase IV genes (e.g., *parC*) [62], as observed in the MDR ciprofloxacin resistant isolate from ground turkey in this study (*gyrA* mutations S82L and D87N). Plasmid-mediated quinolone resistance determinants (e.g., *qnrB19* from an MDR isolate from pork chop with decreased susceptibility to ciprofloxacin in this study) confer low-level resistance but nevertheless still impart concerns due to the reported frequencies of their of co-occurrence with other AMR genetic determinants—for instance, in ESBL-producing *E. coli*—and their high propensity to simultaneously disseminate multiple resistances [63,64,65,66]. To this point, we observed low prevalence of cephalosporin resistant *E. coli* in our study, which was mediated by AmpC-type beta-lactamase gene, *bla*_CMY-2_, but these isolates were MDR with carriage of over eight other AMR genes. 

Previous studies have evaluated the ability of WGS to predict *E. coli* phenotypic resistance, with findings of imperfect but high overall concordance between genotypic and phenotypic resistance [20,67,68], as also observed in this study. The discrepancies in sensitivity for amoxicillin/clavulanic acid and chloramphenicol in our study could be attributed to the very small number of resistant isolates for these drugs which the analysis was based on. Moreover, the concordance of WGS with AMR phenotype is heavily dependent on the categorization of susceptible and resistant isolates, with grouping of intermediate isolates potentially affecting results, alongside any ambiguity in breakpoints used. An example of the latter is the lack of Clinical and Laboratory Standards Institute (CLSI) criteria for streptomycin, a veterinary drug which exhibited both imperfect sensitivity and specificity here and discordance in prior studies [67,69,70,71]. Additionally, AMR genetic determinants conferring less definitive phenotypic resistances such as those encoding multidrug efflux pump genes (e.g., *mdf*(*A*) in this study) add complexity to both data analysis and interpretation. Other considerations have been detailed previously and include the impacts of technical processes in conducting AST/WGS, database selection, and thresholds used for determining the presence or absence of AMR genetic determinants [72]. Lastly, it should be noted that our study evaluated a small number of isolates, all of which were derived from retail meats limited in one geographic region. Thus, while this study finds WGS to be a robust tool for phenotypic AMR predictions in foodborne *E. coli*, its utility for other bacteria from different sources should be considered with caution and supplemented with phenotypic testing to ensure comprehensive AMR assessment. 

*E. coli* isolates in this study were genomically heterogeneous with respect to AMR, with meat type, season, packaging, and label claim accounting for very little of the variability in AMR genetic determinants and a sparsity of shared gene networks observed. Our results suggest that AMR acquisition in *E. coli* from retail meat exhibits greater complexities that could not be fully explained by the retail-level factors assessed and/or by the data collected, as the small number of isolates in this study are likely not representative of the diversity of *E. coli* as a whole from retail meats. Moreover, a limitation of retail level surveillance conducted in this study is that other factors along the farm-to-fork continuum which could potentially contribute to AMR could not be evaluated. Nevertheless, our findings from AMR gene co-occurrence networks reflect certain intricacies of AMR dynamics. For instance, we observe elevated co-occurrence of gene networks corresponding to a broad multidrug transporter (*mdf*(*A*)) and to tetracycline, sulfonamide, and aminoglycoside genes which confer resistance to antimicrobials that are conventionally used in food animal production in the US. The higher frequencies of co-occurrence of these genes (*tet*(*A*), *tet*(*B*), *aph*(*3″*)*-Ib*, *aph*(*6*)*-Id*, *aac*(*3*)*-VIa*, *ant*(*3″*)*-Ia*, *sul1*, and *sul2*) could result from direct exposure to the corresponding antimicrobials at some point along the food chain, or persistence as a result of co-selection from these genes occurring on the same mobile genetic element (e.g., plasmids). We did not assess AMR gene carriage on plasmids in this study due to the limited capacity of short-read sequencing data to fully resolve plasmid structures [73]; however, other studies employing long-read sequencing have confirmed the occurrence of these genes on the same plasmid(s) in *E. coli* [74,75,76]. Lastly, the diversity of AMR genetic determinants identified in this study—including those corresponding to antimicrobials not used in food animals in the US (e.g., *mcr-9*)—suggests that the accumulation of AMR reservoirs could occur even in the absence of direct selective pressures, with the acquisition and loss of certain AMR genes in *E. coli* possibly attributed to the presence or absence of fitness costs that are associated with the maintenance of these genes [77,78].

## 4. Materials and Methods

### 4.1. Study Area and Sampling

Samples in this study were collected as part of routine NARMS retail meat surveillance in 2018, when the program expanded geographical coverage to include southern California. From January to December 2018, a total of 480 fresh retail meat samples consisting of 240 skin-on/bone-in chicken, 120 ground turkey, 60 ground beef, and 60 pork chops were purchased from retail grocery stores in southern California twice each month. Sampling locations were selected based on the NARMS retail store sampling plan through random selection of grocery stores within zip codes corresponding to West Los Angeles, East Los Angeles, Ontario, and Irvine. Samples were transported on ice to the laboratory, refrigerated, and processed within 72 h of purchase. 

### 4.2. Sample Processing and E. coli Isolation

A random selection of 221 samples (56 chicken, 54 ground turkey, 55 ground beef, and 56 pork chops) was processed for isolation of *E. coli* per the 2018 NARMS Retail Meat Surveillance protocol [79]. Briefly, 25 g of each sample was placed in Whirl-Paks containing 250 mL buffered peptone water (Becton Dickinson, Franklin Lakes, NJ, USA) and hand massaged for 3 min. A total of 50 mL of rinsate was then added to 50 mL double-strength MacConkey broth (Becton Dickinson, Franklin Lakes, NJ, USA) and incubated at 35 °C for 24 h. Following overnight enrichment, a loopful (10 µL) was streaked to a MacConkey plate and incubated at 35 °C for 24 h. One suspect *E. coli* colony based on typical colony morphology was streaked to purity on blood agar plates and incubated overnight at 35 °C. Isolates were confirmed as *E. coli* using biochemical tests (indole positive and oxidase negative), and banked in Brucella broth with 15% glycerol, frozen, and shipped on dry-ice to the FDA’s Center for Veterinary Medicine (CVM) for antimicrobial susceptibility testing and whole-genome sequencing. 

### 4.3. Antimicrobial Susceptibility Testing

*E. coli* isolates were tested using a broth microdilution method for 14 antimicrobial drugs using the NARMS Gram-negative plates (Thermo Fisher Scientific, Waltham, MA, USA) per standard protocols [80]. NARMS breakpoints were used to classify isolates into susceptible, intermediate, and resistant categories based on the minimum inhibitory concentration (MIC) values for each drug; these breakpoints are based on the CLSI guidelines with the exception of streptomycin and azithromycin, where NARMS consensus interpretive criteria were used due to absence of available CLSI breakpoints for these two drugs (Table S2) [81]. Due to the limited range of dilutions in the drug panel, resistance could not be determined for azithromycin and sulfisoxazole and only susceptible classification were determined for these two drugs. For analysis, intermediate and susceptible isolates were grouped together. Decreased susceptibility (DSC) to ciprofloxacin (≥0.12 µg/mL) was also noted in descriptive analyses due to the expanded definition from CLSI for its intermediate susceptibility MIC range [81]. Multidrug resistance was defined as resistance to one or more drugs in three or more antimicrobial classes [82].

### 4.4. Whole-Genome Sequencing and Identification of Resistance Genes

A subset of *E. coli* isolates (n = 52) was randomly selected to undergo whole-genome sequencing (WGS) by short-read sequencing on the Illumina MiSeq using v2 or v3 chemistry for 2 × 250-bp paired end reads, and identification of resistance genes was conducted as previously described [67]. Briefly, genomic DNA was extracted using the DNeasy Blood and Tissue kit (Qiagen, Valencia, CA, USA), and libraries were prepared using the Illumina Nextera XT kit per manufacturer’s protocols. Sequences were demultiplexed using MiSeq Reporter and assembled using the CLC Genomics Workbench. The ResFinder database (Center for Genomic Epidemiology, DTU) was used to identify resistance gene hits (≥85% amino acid identity and ≥50% sequence length), and Perl scripts were used to extract and analyze the *gyrA* gene at amino acid position 83 and 87 to assess chromosomal mutations associated with quinolone resistance. 

### 4.5. Data Analysis

Descriptive statistics (prevalence of *E. coli*, distribution of predictor variables, antimicrobial susceptibility testing results, and prevalence of antimicrobial resistance genes) and binary logistic regression models were conducted using SAS On-Demand for Academics. Predictor variables evaluated in this study include meat type (ground turkey, chicken, pork chop, and ground beef), time of year of sample purchase, packaging type (modified atmosphere packaging, plastic film, vacuum, chub, paper, and plastic bag), label claims, packaging type, and presence of *Salmonella*. For label claims, reduced antibiotic use included samples labeled organic or reduced/no antibiotic usage; all other samples with absence of such label claims were classified as conventional. Retail meat samples in this study were concurrently processed for isolation of *Salmonella*, with data on the presence of *Salmonella* obtained through methods detailed previously [69].

The association between the presence of *E. coli* in retail meat samples and predictor variables were evaluated using logistic regression models. Univariable logistic regression models were used to evaluate the crude association between each predictor variable and the outcome binary variable, which was designated as the presence or absence of a recovered *E. coli* isolate from the retail meat sample. A multivariable logistic regression model was then fitted based on the retention of significant variables, assessment of collinearity, testing of all two-way interactions, and best model fit as determined by the lowest Akaike’s Information Criterion (AIC). The association between predictor variables and whether an *E. coli* isolate from retail meat was multidrug resistant or not, and whether it was resistant or not to ampicillin, gentamicin, streptomycin, and tetracycline, were individually evaluated using exact logistic regression models. These four drugs were selected for evaluation due to the higher frequency of observed resistance.

Prediction of phenotypic antimicrobial resistance (AMR) from antimicrobial susceptibility testing based on genotypic AMR from the presence of antimicrobial resistance genes was evaluated as previously described [69]. Briefly, phenotype and genotype concordance for each drug included true positives (TP: resistant isolate with corresponding AMR genetic determinants) and true negatives (TN: susceptible isolates with absence of corresponding AMR genetic determinants). Discordance included false negatives (FN: resistant isolates with absence of corresponding AMR genetic determinants) and false positives (FP: susceptible isolates with presence of corresponding AMR genetic determinants). Sensitivity and specificity were then calculated as TP/(TP+FN) and TN/(TN+FP), respectively. Due to the absence of phenotypically resistant isolates to sulfisoxazole, azithromycin, and meropenem, sensitivity could not be assessed for them, so these three drugs were omitted from the overall calculation for sensitivity. Multidrug resistance gene, *mdf(A)*, was omitted from the concordance analysis due to ambiguity in its AMR phenotype conferral. 

To assess the collective AMR gene profiles of *E. coli* isolates, clustering based on the presence and absence of AMR genetic determinants was evaluated using functions in the vegan package [83] in R by grouping factors of meat type, season of retail meat purchase, packaging type, and label claim. Non-metric multidimensional scaling (NMDS) was performed using the metaMDS function with a Jaccard distance metric and in two dimensions. The permutest and betadisper functions were used to conduct a PERMDISP2 procedure to evaluate if dispersions of groups for each of the grouping factors were homogenous [84,85]. Permutational analysis of variance (PERMANOVA) was conducted to test the equivalence of centroids of groups for each grouping factor using the adonis2 function. Additionally, analysis of similarity (ANOSIM) was performed to evaluate for each grouping factor whether the average of ranks within-group distances was greater or equal to that of between-group distances [38]. The aforementioned tests (PERMDISP2, PERMANOVA, and ANOSIM) were performed using 10,000 permutations and a Jaccard distance metric. 

From the presence and absence data of AMR determinants in *E. coli* isolates, a pairwise co-occurrence matrix was constructed by transforming the binary data. The resulting co-occurrence data were then visualized as networks of co-occurring genes using Gephi [86], with nodes representing genes and edges representing the frequency of co-occurrence. Networks were evaluated by frequency of co-occurrence thresholds based on ≥1 genome (all *E. coli* isolates), ≥5 genomes, and ≥10 genomes.

## Figures and Tables

**Table 1:** Risk factors for presence of *E. coli* in retail meat products from southern California.

Risk Factor		Univariable Models		Multivariable Model	
	*E. coli* Positive n/N (%)	OR (95% CI)	*p*-Value	OR (95% CI)	*p*-Value
Meat Type					
Chicken	38/56 (67.86%)	8.44 (3.55, 20.09)	<0.0001	9.43 (3.84, 23.21)	<0.0001
Ground Turkey	38/54 (70.37%)	9.50 (3.93, 22.95)	<0.0001	11.00 (4.39, 27.54)	<0.0001
Pork Chop	18/56 (32.14%)	1.90 (0.80, 4.51)	0.15	1.93 (0.79, 4.71)	0.15
Ground Beef	11/55 (20.00%)	1.00	-	1.00	-
Season					
Spring	22/60 (36.67%)	0.30 (0.13, 0.69)	0.0046	0.22 (0.083, 0.55)	0.0015
Summer	28/60 (46.67%)	0.45 (0.20, 1.03)	0.0592	0.36 (0.14, 0.91)	0.031
Autumn	28/60 (46.67%)	0.45 (0.20, 1.03)	0.0592	0.36 (0.14, 0.91)	0.031
Winter	27/41 (65.85%)	1.00	-	1.00	-
Packaging type					
Modified atmosphere packaging (MAP)	55/85 (64.71%)	3.40 (1.55, 7.48)	0.0023	-	-
Plastic film	26/64 (40.63%)	1.27 (0.56, 2.88)	0.57	-	-
Other (Vacuum, chub, paper)	10/32 (31.25%)	0.84 (0.31, 2.27)	0.74	-	-
Plastic bag	14/40 (35.00%)	1.00	-	-	-
Label Claim					
Reduced antibiotic claim	27/37 (72.97%)	3.67 (1.68, 8.02)	0.0011	-	-
Conventional	78/184 (42.39%)	1.00	-	-	-
Presence of *Salmonella*					
Yes	8/11 (72.73%)	3.11 (0.80, 12.04)	0.10	-	-
No	97/210 (46.19%)	1.00	-	-	-
Overall prevalence	105/221 (47.51%)	-	-	-	-

**Table 2:** Distribution of phenotypic antimicrobial resistance in *E. coli* isolates from retail meat samples (n = 105).

		Number of Isolates Resistant to Antimicrobial Drugs (%)				
CLSI Class	Antimicrobial Agent	Chicken (n = 38)	Ground Turkey (n = 38)	Pork Chop (n = 18)	Ground Beef (n = 11)	All Samples (n = 105)
Aminoglycosides	STR	10 (26.32%)	17 (44.74%)	5 (27.78%)	0 (0%)	32 (30.48%)
	GEN	9 (23.68%)	7 (18.42%)	1 (5.56%)	0 (0%)	17 (16.19%)
Beta-lactam combination agents	AMC	1 (2.63%)	2 (5.26%)	0 (0%)	0 (0%)	3 (2.86%)
Cephems	FOX	1 (2.63%)	1 (2.63%)	0 (0%)	0 (0%)	2 (1.90%)
	AXO	1 (2.63%)	1 (2.63%)	0 (0%)	0 (0%)	2 (1.90%)
Folate pathway antagonists	COT	0 (0%)	2 (5.26%)	0 (0%)	0 (0%)	2 (1.90%)
Macrolides	AZI	0 (0%)	0 (0%)	0 (0%)	0 (0%)	0 (0%)
Penems	MER	0 (0%)	0 (0%)	0 (0%)	0 (0%)	0 (0%)
Penicillins	AMP	5 (13.16%)	14 (36.84%)	3 (16.67%)	0 (0%)	22 (20.95%)
Phenicols	CHL	0 (0%)	2 (5.26%)	0 (0%)	0 (0%)	2 (1.90%)
Quinolones	NAL	0 (0%)	1 (2.63%)	1 (5.56%)	0 (0%)	2 (1.90%)
	CIP	0 (0%)	1 (2.63%)	0 (0%)	0 (0%)	1 (0.95%)
Tetracycline	TET	15 (39.47%)	24 (63.16%)	7 (38.89%)	0 (0%)	46 (43.81%)

STR: streptomycin; GEN: gentamicin; AMC: amoxicillin/clavulanic acid; FOX: cefoxitin; AXO: ceftriaxone; COT: trimethroprim-sulfamethoxazole; AZI: azithromycin; MER: meropenem; AMP: ampicillin; CHL: chloramphenicol; NAL: nalidixic acid; CIP: ciprofloxacin; TET: tetracycline.

**Table 3:** Association between retail meat type and phenotypic antimicrobial resistance.

Meat Type	Antimicrobial Drug (Abbreviation)							
	Ampicillin (AMP)		Gentamicin (GEN)		Streptomycin (STR)		Tetracycline (TET)	
	OR (95% CI)	*p*-Value	OR (95% CI)	*p*-Value	OR (95% CI)	*p*-Value	OR (95% CI)	*p*-Value
Chicken	1.31 (0.23, 9.20)	1.00	8.47 (1.05, 394.18)	0.042	1.70 (0.45, 7.27)	0.56	2.03 (0.63, 7.08)	0.29
Ground turkey	4.94 (1.17, 30.11)	0.025	6.18 (0.72, 294.92)	0.13	3.81 (1.10, 15.56)	0.032	5.24 (1.65, 18.55)	0.003
Non-poultry (beef, pork)	1.00	-	1.00	-	1.00	-	1.00	-

**Table 4:** Distribution of phenotypic antibiogram patterns in *E. coli* isolates (n = 105).

Antibiogram Pattern	Number of Isolates (n/N %)	Multidrug Resistant
Susceptible to all drugs in MIC panel	51 (48.57%)	No
TET	12 (11.43%)	No
STR-TET	8 (7.62%)	No
AMP-TET	5 (4.76%)	No
GEN-STR-TET	5 (4.76%)	No
GEN-STR	4 (3.81%)	No
AMP	2 (1.90%)	No
AMP-GEN-STR-TET	2 (1.90%)	Yes
AMP-GEN-TET	2 (1.90%)	Yes
AMP-STR-TET	2 (1.90%)	Yes
AMC-AMP-FOX-AXO-GEN-STR-TET	1 (0.95%)	Yes
AMC-AMP-FOX-AXO-GEN-TET	1 (0.95%)	Yes
AMC-AMP-STR-TET	1 (0.95%)	Yes
AMP-CHL-STR-TET	1 (0.95%)	Yes
AMP-CHL-STR-TET-COT	1 (0.95%)	Yes
AMP-GEN-NAL-STR-TET-CIP	1 (0.95%)	Yes
AMP-GEN-STR	1 (0.95%)	No
AMP-NAL-STR-TET-CIP^dsc^ *	1 (0.95%)	Yes
AMP-STR-TET-CIP^dsc^ *	1 (0.95%)	Yes
STR	1 (0.95%)	No
STR-TET-CIP^dsc^	1 (0.95%)	No
STR-TET-COT	1 (0.95%)	Yes

* CIP^dsc^ denotes decreased susceptibility to ciprofloxacin. STR: streptomycin; GEN: gentamicin; AMC: amoxicillin/clavulanic acid; FOX cefoxitin: AXO ceftriaxone; COT: trimethroprim-sulfamethoxazole; AZI: azithromycin: MER: meropenem; AMP: ampicillin; CHL: chloramphenicol; NAL: nalidixic acid; CIP: ciprofloxacin; TET: tetracycline.

**Table 5:** Results of PERMDISP2, PERMANOVA, and ANOSIM tests based on the presence and absence of AMR genetic determinants. All tests were performed using a Jaccard distance metric and 10,000 permutations.

Grouping Factor	PERMDISP2 *p*-Value (F)	PERMANOVA *p*-Value (R^2^)	ANOSIM *p*-Value (R)
Meat type	0.010 (4.25)	0.10 (0.088)	0.89 (−0.059)
Season	0.41 (0.97)	0.73 (0.044)	0.80 (−0.022)
Packaging type	0.78 (0.36)	0.55 (0.053)	0.24 (0.038)
Label claim	0.39 (0.78)	0.80 (0.0094)	0.87 (−0.11)

## Data Availability

Whole-genome sequences of *E. coli* isolates in this study are deposited under BioProject PRJNA292663.

## Supplementary Materials

The following supporting information can be downloaded at: https://www.mdpi.com/article/10.3390/antibiotics12040782/s1, Table S1: List of *E. coli* isolates in this study; Table S2: NARMS breakpoints used to classify minimum inhibitory concentrations of *E. coli* isolates; Table S3: Genotypic prediction of phenotypic resistance in *E. coli* isolates from retail meat (n = 52).
